# Vulvar Cancer with Cutaneous and Breast Metastases

**DOI:** 10.1155/2021/8241815

**Published:** 2021-01-18

**Authors:** Nawaraj Adhikari, Adarsh Vennepureddy, Sara Parylo, Anupma Agarwal, Meekoo Dhar

**Affiliations:** ^1^Department of Medicine, Staten Island University Hospital, 475 Seaview Avenue, Staten Island, NY 10305, USA; ^2^Department of Haematology/Oncology, Staten Island University Hospital, 475 Seaview Avenue, Staten Island, NY 10305, USA; ^3^Department of Pathology and Laboratory Medicine, Staten Island University Hospital, 475 Seaview Avenue, Staten Island, NY 10305, USA

## Abstract

Vulvar cancer accounts for about 5% of cancer of female genitalia. It may initially present as benign symptoms resulting in potential delay in diagnosis. Few cases of distant metastases to skin or breast have been reported. We present the case of a 76-year-old female with possible delay in diagnosis of her squamous cell carcinoma of vulva. After 4 months of the diagnosis, she presented with concurrent cutaneous and breast metastases.

## 1. Introduction

There were estimated 6120 new cases of vulvar cancer in the US in the year 2020, accounting for about 5% of cancer of female genitalia and 0.6% of all cancers in women [1]. The median age at diagnosis of vulvar cancer is 68 years [2]. Vulvar cancer may initially present with nonspecific symptoms like pruritus, pain, burning sensation, bleeding, and lumps. This may be diagnosed as inflammation of Bartholin's gland or inflammation, atrophy, or hypertrophy of vulva which may delay the diagnosis of vulvar cancer. Vulvar cancer may also arise from preexisting, known disease, like lichen sclerosus, and identifying their evolution towards tumor may be difficult, especially in early stages. Squamous cell carcinoma (SCC) accounts for about 90% of vulvar carcinoma. SCC usually presents as localized disease (59%); however, 30% presents with spread to the regional lymph node, 6% with distant metastases, and 5% unstaged [2]. Our PubMed-based search revealed that only 3 cases of vulvar carcinoma metastasis to breast and 16 cases of metastases to skin have been documented prior to our case report. Here, we present a case of squamous cell carcinoma of vulva with potential delay in diagnosis, which after 4 months of diagnosis presented with concurrent metastases to breast and skin.

## 2. Case Description

**Figure 1:**
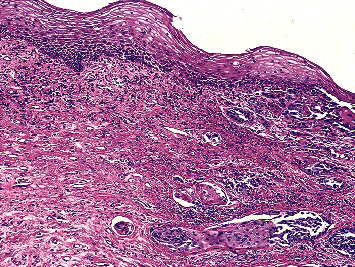
Vulvar mass showing invasive squamous cell carcinoma (H and E, ×100).

A 76-year-old female with a past medical history of coronary artery disease status postcoronary artery bypass graft, hypertension, and aortic stenosis status postmetallic aortic valve replacement presented to the emergency department, with intense vaginal pain and bleeding for three weeks. She had history of 30 pack-years smoking in the past. She had presented to gynaecology clinic about 9 months back with mild vaginal itching and spotting. She was initially given clotrimazole/betamethasone cream which she did not tolerate because of burning sensation and then was given topical oestrogen ointment with partial resolution of her symptoms. A transvaginal ultrasound showed an endometrial strip of 5 mm, but no mass was visualized. She was planned for hysteroscopy and dilatation and curettage if symptoms persist; however, there was no follow-up with the gynaecology clinic for 7 months prior to this emergency department visit. Currently, her pelvic examination revealed midline clitoral mass with erythematous foul-smelling discharge; however, examination was limited due to severe tenderness. Rest of the physical examination was unremarkable. Examination under anaesthesia showed 4.5 cm clitoral mass encompassing right and left labia minora, which was indurated and erythematous. Biopsy of the mass was compatible with invasive squamous cell carcinoma (Figure 1). The neoplastic cells were positive for CK5/6, p63, AE1/AE3, cam5.2, CK7, and CK34betaE12 while negative for p16, CK20, PR, GCDFP15, HMB-45, Mart 1, PASD, and mucicarmine. CEA-P and S100 showed focal staining. PET CT revealed a focus of increased radiotracer uptake within the region of vaginal orifice and no other focal area of intake. Based on these findings, diagnosis of invasive squamous cell carcinoma was made. Given the comorbidities and extent of disease, she was treated with primary radiotherapy to vulva and pelvis which was completed over next 3 months.

**Figure 2:**
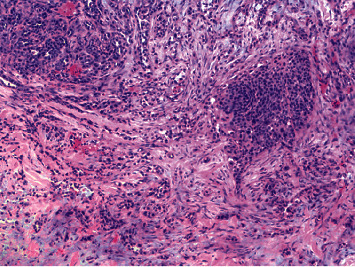
Left upper back mass—metastatic squamous cell carcinoma (H and E, ×100).

**Figure 3:**
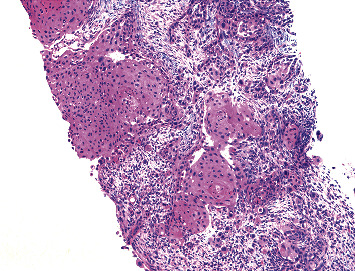
Right breast mass—metastatic squamous cell carcinoma (H and E, ×100).

During her regular follow-up 4 months after the diagnosis, she reported improvement of vaginal pain but was found to have a rapidly growing 7 × 5 cm mass palpable in the left upper back. The skin overlying the mass was normal and intact. PET CT showed near complete resolution of uptake in the region of vulva, focal uptake in the region of left inguinal lymph node, right breast, and in the soft tissue lesion of left upper back. Core biopsy of the left upper back lesion was done (Figure 2), and the immunostaining profile was similar to prior vulvar mass biopsy specimen suggesting metastasis from vulva. Ultrasound-guided core needle biopsy of right breast mass (Figure 3) demonstrated invasive squamous cell carcinoma with focal necrosis. The immunophenotype was consistent with metastasis from vulvar primary and back mass metastasis.

She was started on carboplatin and paclitaxel chemotherapy. She received the first cycle of chemotherapy. Her second cycle of chemotherapy was delayed because she was admitted to hospital for pneumonia. After discharge from hospital, she opted for hospice and succumbed to her illness in 9 months from the initial diagnosis of vulvar cancer.

## 3. Discussion

The most common symptom of vulvar cancer is prolonged history of pruritus, followed by vulvar bleeding, dysuria, discharge, and vaginal pain [3]. Clinicians should opt for vulvar skin biopsy when there are persistent symptoms as valvular itching or there is valvular lesion of uncertain significance. A retrospective study done in 1652 women from 218 gynaecological practice in Germany showed a potential mean delay of diagnosis of 186–328 days [4]. In our case, there was a lag period of around 7 months, when she was last seen in outpatient clinic to her hospitalization when eventually the diagnosis was made. This could have resulted in potential delay in the diagnosis.

Metastasis of vulvar cancer occurs via local spread, lymphatic system, and haematogenous pathway. Lymphatics from vulva mainly drain into the superficial inguinal nodes and then into the deep inguinal lymph nodes and follows the iliac vasculature to the external iliac nodes and ultimately the para-aortic nodes. Lymphatics from clitoris can sometimes proceed directly to the deep inguinal lymph nodes or less commonly to the external iliac nodes [5].

In a retrospective study done in a gynaecological centre in Germany, between 1996 and 2013, 391 patients with primary squamous cell carcinoma were treated, out of which 20 patients (5.1%) had distant metastases, and reported sites were lung, liver, bone, skin, and lymph nodes [6]. Our PubMed-based search revealed only 3 cases of breast metastases reported, out of which 2 were unilateral and 1 was bilateral [7–9]. There were 16 cutaneous metastases reported in thigh, abdomen, left lower back, forearm, buttock, and groin [10]. Our patient presented with concurrent breast and cutaneous metastases 4 months after the diagnosis of vulvar carcinoma. The 5-year relative survival rate for localized vulvar cancer (without spread to lymph node or nearby tissues) is 86%, regional disease (spread to nearby lymph nodes or tissue but not to distant organs) is 53%, and distant spread (spread to lungs, liver, bone, and breast) is 19% [11]. Distant metastases have a very poor prognosis, and treatment is mainly palliative which may include chemotherapy, radiation, or surgery for comfort.

## 4. Conclusion

Vulvar cancer may initially present as benign appearing symptoms and clinicians dealing with this especially in elderly should monitor closely for an early diagnosis. Vulvar cancer patients during their follow-up should be carefully assessed for distant metastases.
